# Histological analysis of psoriasiform eruption associated with myelin oligodendrocyte glycoprotein antibody-associated disease

**DOI:** 10.1016/j.jdcr.2023.12.026

**Published:** 2024-02-10

**Authors:** Saori Itoi-Ochi, Yukiho Kurosaki, Asako Ota, Akiko Miyazaki, Akiko Hosokawa, Misa Nakano, Toshiyuki Takahashi, Noriko Umegaki-Arao, Manabu Fujimoto

**Affiliations:** a Department of Dermatology, Suita Municipal Hospital, Suita, Japan; b Department of Neurology, Suita Municipal Hospital, Suita, Japan; c Department of Neurology, Tohoku University Graduate School of Medicine, Sendai, Japan; d Department of Neurology, National Hospital Organization Yonezawa National Hospital, Yonezawa, Japan; e Department of Dermatology, Tokyo Women’s Medical University, Adachi Medical Center, Tokyo, Japan; f Department of Dermatology, Osaka University Graduate School of Medicine, Suita, Osaka, Japan

**Keywords:** CD20-positive lymphocytes, CD68-positive macrophages, myelin oligodendrocyte glycoprotein antibody-associated disease, skin eruption

## Introduction

Myelin oligodendrocyte glycoprotein (MOG) is located on the surface of myelin and is mainly expressed in the central nervous system.^1^ Oligodendrocyte glycoprotein antibody-associated disease (MOGAD) is a recently identified autoimmune disease that causes inflammatory demyelination, including optic neuritis and encephalomyelitis.^2^ However, associated skin findings in MOGAD have not been reported.^3^ Here, we report a psoriasiform eruption in the setting of MOGAD, including histological analysis with a comparative assessment to psoriasis lesions in the same patient.

## Case report

**Fig 1:**
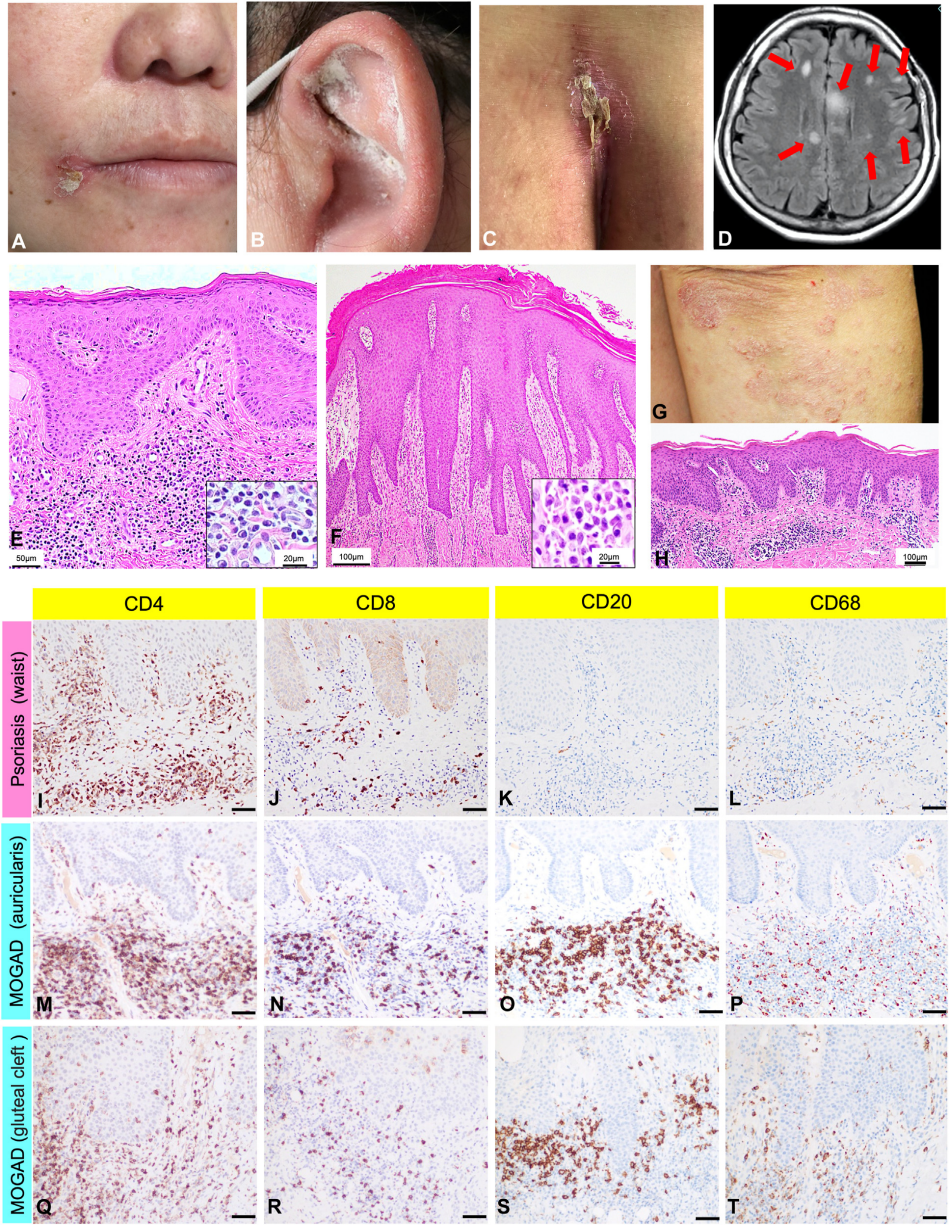
Clinical, radiologic, histopathological, and immunohistochemical characteristics of the patient. **A** to **C,** Scaly erythematous plaques on the tip of the nose, corners of the mouth (**A**), external ear (**B**), and gluteal cleft (**C**). **D,** Brain magnetic resonance imaging reveals multiple hyperintense lesions on fluid-attenuated inversion recovery images (indicated by the *red arrows*) that are displayed on gadolinium-enhancing T1-weighted images as well. **E** and **F,** A skin biopsy of the scaly erythematous lesions on the external ear (**E**) and the gluteal cleft (**F**) revealed hyperkeratosis and thickening of the epidermis, no loss of the granular layer, and infiltration of numerous plasma cells and lymphocytes in the upper to middle layers of the dermis [hematoxylin and eosin staining; bar = (**E**) 50 μm, (**F**) 100 μm; inset bar = 20 μm]. **G** and **H,** Clinical and histopathologic findings of the rash diagnosed as psoriasis 12 years prior to the first. **G,** The skin eruptions are scattered in a disseminated manner on the head, trunk, and extremities. **H,** Scaly erythematous lesions reveal epidermal thickening and perivascular lymphocyte infiltration in the upper dermis (hematoxylin and eosin staining; bar = 100 μm). **I** to **T,** Immunohistochemical analysis of the scaly erythematous lesions using a rabbit monoclonal anti-CD4 (SP35) antibody (Roche, 790-4423) at 1:200 dilution, a rabbit monoclonal anti-CD8 (SP57) antibody (Roche, 790-4460) at 1:200 dilution, a mouse monoclonal anti-CD20 (L26) antibody (Roche, 760-2531), and a mouse monoclonal anti-CD68 (PG-M1) antibody (Dako, M 0876) at 1:100 dilution (bar = 50 μm). *MOGAD*, Myelin oligodendrocyte glycoprotein antibody-associated disease.

A 47-year-old Japanese woman presented with acute progressive decreased attention, gait disturbance, dysuria, and skin rash. She had a history of psoriasis, currently in remission. Clinical findings at the initial visit revealed scaly erythema on the tip of the nose, corners of the mouth, external ear, and gluteal cleft (Fig 1, *A*-*C*). Blood tests revealed a lymphocyte count of 590/μL (normal 1000-4800), C3 level of 63 mg/dL (normal 75-175), and IgG level of 2466 mg/dL (normal 600-1600). Serum IgG4, anti-double-stranded DNA antibodies, and other autoimmune markers tested were negative. Urinalysis revealed transient proteinuria, but renal biopsy findings were inconsistent with lupus nephritis. Magnetic resonance imaging of the brain and spinal cord demonstrated multiple gadolinium-enhancing lesions in the cerebral white matter, brain stem, and spinal cord located in C3 (C: cerrical spine), C7, and Th3 (Th: thoracic) (Fig 1, *D*). Cerebrospinal fluid (CSF) examination revealed an elevated cell count with mononuclear cell predominance (38/μL). CSF protein and myelin basic protein levels were elevated at 61 mg/dL and >2000 pg/mL, respectively, but oligoclonal bands were negative. Serum and CSF MOG antibodies were increased 128- and 32-fold, respectively, leading to a diagnosis of MOGAD (serum low positive, CSF positive, and supporting brain clinical/magnetic resonance imaging features).^2^ Skin biopsies, taken from the scaly erythema on the external ear and gluteal region, demonstrated hyperkeratosis and thickening of the epidermis, no loss of the granular layer, and infiltration of numerous plasma cells and lymphocytes in the upper to middle layers of the dermis (Fig 1, *E* and *F*). Direct immunofluorescence analysis was negative for IgG, IgA, IgM, and C3. To distinguish the skin eruption associated with MOGAD from her prior psoriasis lesions (Fig 1, *G*), we performed an immunohistochemical analysis. The skin biopsy taken from psoriasis 14 years ago showed hyperkeratosis with parakeratosis, regular elongation of rete ridges, loss of granular layer, and perivascular inflammatory cell infiltration (Fig 1, *H*). Immunohistologically, CD4-dominant T cells intensively infiltrated the perivascular region of the upper dermis, but very few CD20-positive B cells and CD68-positive macrophages were observed (Fig 1, *I*-*L*), which were considered typical for psoriasis.^4^ In the skin eruption associated with MOGAD on the external ear, CD4- and CD8-positive T cells, CD20-positive B cells, and CD68-positive macrophages briskly infiltrated the upper dermis (Fig 1, *M*-*P*). Similar findings were identified in skin biopsy from the intergluteal cleft (Fig 1, *Q*-*T*).

After methylprednisolone pulse therapy, oral prednisolone (40 mg/d) resolved the clinical finding of encephalomyelitis and all the lesions of scaly erythema. Prednisolone tapering to 12 mg/d caused no flare in skin findings.

## Discussion

Recently, several studies reported that MOG functions as an essential target for autoantibodies and cell-mediated immune responses in MOGAD.^5^, ^6^, ^7^, ^8^ Previous studies have suggested that Th1 and Th17 cells are involved in autoimmune encephalomyelitis.^5^^,^^6^ In a case report, immunohistochemical analysis of brain biopsy specimens in MOGAD has revealed that CD3-positive T cells, CD20-positive B cells, and CD68-positive macrophages infiltrate around blood vessels.^7^ In addition, Takai et al conducted a brain biopsy on 11 cases of MOGAD, confirming infiltrated cells consisting mainly of CD4-dominant T cells, CD20-positive B cells, and CD68-positive macrophages in the perivascular space and demyelinating lesions. They concluded that CD4-predominant T-cell infiltration and perivascular macrophages are the characteristic findings of the acute phase of MOGAD, which is different from other demyelinating diseases.^8^ In our case, the patient presented with scaly erythema clinically indistinguishable from psoriasis. However, the distribution of her current lesions was different from those of her prior episodes of psoriasis. The histological and immunohistochemical analysis in our patient showed hyperkeratosis and parakeratosis with perivascular infiltrations of CD4-positive T cells in both the skin eruption associated with MOGAD and psoriasis, which would support the similarity in clinical appearance. Interestingly, we confirmed the presence of significant CD20-positive B cells and CD68-positive macrophages in the dermis in the lesions associated with MOGAD, consistent with the findings noted in brain biopsies of MOGAD previously reported. Because MOG protein expression is limited to the central nervous system,^1^ it would be unclear which molecules in the skin would induce the immune response in our MOGAD case. To the best of our knowledge, there are no previous reports of skin findings associated with MOGAD. Therefore, it is unclear whether skin eruption occurs in MOGAD patients with or without a history of psoriasis. However, considering the immunohistochemical findings of MOGAD demyelinating lesions by brain biopsy, the CD20-positive B cell and CD68-positive macrophage infiltration into the skin, as well as CD4-positive T cells, could be the trigger for psoriasis-like scaly erythema.

In the future, additional reports of MOGAD-related cutaneous finding may help to clarify the mechanisms and characteristics of MOGAD-related skin eruption.

## Conflict of interest

None disclosed.
